# Inflammation-scores as prognostic markers of overall survival in lung cancer: a register-based study of 6,210 Danish lung cancer patients

**DOI:** 10.1186/s12885-021-09108-5

**Published:** 2022-01-14

**Authors:** Anne Winther-Larsen, Ninna Aggerholm-Pedersen, Birgitte Sandfeld-Paulsen

**Affiliations:** 1 Department of Clinical Biochemistry, Aarhus University Hospital, Aarhus, Denmark; 2 Department of Oncology, Aarhus University Hospital, Aarhus, Denmark; 3 Department of Clinical Biochemistry, Viborg Regional Hospital Heibergs Allé 5A8800, Viborg, Denmark

**Keywords:** Lung cancer, Inflammation-score, Neutrocyte-lymphocyte-raio (NLR), Modified Glasgow Prognostic Score (mGPS), Aarhus Composite Biomarker Score (ACBS), Combined NLR and GPS (CNG)

## Abstract

**Background:**

Inflammation-scores based on general inflammation markers are suggested as prognostic markers of overall survival (OS) in lung cancer. However, whether these inflammation-scores improves the prognostication performed by well-established prognostic markers is unsettled. In a large register-based lung cancer patient cohort, nine different inflammation-scores were compared, and their ability to optimize the prognostication of OS was evaluated.

**Methods:**

Lung cancer patients diagnosed from 2009–2018 in The Central Denmark Region were identified in the Danish Lung Cancer Registry. Pre-treatment inflammation markers were extracted from the clinical laboratory information system. Prognostication of OS was evaluated by Cox proportional hazard models. Comparison of the inflammation-scores and their added value to established prognostic markers were assessed by Akaike's information criteria and Harrel's C-index.

**Results:**

In total, 5,320 patients with non-small cell lung cancer (NSCLC) and 890 patients with small cell lung cancer (SCLC) were identified. In NSCLC, the Aarhus composite biomarker score (ACBS), including albumin, C-reactive protein, neutrophil count, lymphocyte count and haemoglobin, and the neutrophil-lymphocyte-ratio (NLR) were superior. Furthermore, they improved the prognostication of OS significantly (*p *<0.0001) (ACBS: HR: 2.24 (95%CI: 1.97–2.54); NLR: HR: 1.58 (95%CI: 1.47 – 1.69)). In SCLC, three scores were equally superior and improved the prognostication of OS* p* < 0.0001): neutrophil–lymphocyte-ratio (HR:1.62 (95%CI: 1.38–1.90)), modified Glasgow Prognostic Score (mGPS) (HR:1.70 (95%CI: 1.55–1.86) and the Combined NLR and GPS (CNG) (HR:2.10 (95%CI: 1.77–2.49).

**Conclusions:**

The ACBS was the optimal score in NSCLC, whereas neutrophil–lymphocyte-ratio, mGPS and CNG were equally superior in SCLC. Additionally, these inflammation-scores all optimised the prognostication of OS and added value to well-established prognostic markers.

**Supplementary Information:**

The online version contains supplementary material available at 10.1186/s12885-021-09108-5.

## Introduction

Lung cancer remains the leading cause of cancer-related death [1, 2]. To improve lung cancer survival, we need prognostic markers to identify patients at high risk of inferior survival. Several prognostic markers have been suggested in lung cancer; however, only very few have proven clinically relevant. The tumor, node and metastasis (TNM) staging system, which classifies patients into clinical and pathological stages [3] and the Eastern cooperative oncology group performance status (ECOG PS), which is based on daily life activities [4], are the most well-established prognostic markers in lung cancer. However, the overall survival (OS) varies within each stage and performance status category, and new prognostic markers that can add value to the established prognostic markers are needed.

Inflammation is recognised as one of the hallmarks of cancer as the tumor-associated inflammatory response in the tumour microenvironment leads to tumorigenesis and progression [5]. Therefore, general inflammation markers like C-reactive protein (CRP), leucocytes and lymphocytes have been suggested as prognostic biomarkers in cancer [6–8]. Though, results have been conflicting, which undoubtedly reveals that the individual inflammation marker does not fully reflect the inflammation system. Instead, Inflammation-scores as the neutrophil-to-lymphocyte ratio (NLR), platelet-to-lymphocyte ratio (PLR), and Glasgow Prognostic Score (GPS), based on more than one inflammation marker, have been suggested as optimised biomarkers of the inflammatory system and have demonstrated potential prognostic capacity in different cancers [8–14].

In lung cancer, several inflammation-scores have been evaluated. Especially, NLR, PLR and GPS have attracted attention and in meta-analyses demonstrated potentials as prognostic biomarkers independently of stage and histology [15–19]. However, despite many studies on inflammation-scores in the literature, it remains unknown which inflammation-score serves as the best prognostic score. Furthermore, when new prognostic inflammation-scores are recommended, it is seldom evaluated whether these prognostic markers actually add value to well-established prognostic markers.

Based on a comprehensive literature search, we identified nine inflammation-scores composed of general inflammation markers which have been previously evaluated in lung cancer patients [19]. Our primary objective was to make a direct comparison of the prognostic potential of these established inflammation-scores in a large Danish lung cancer cohort to identify the inflammation-score with the best prognostication of OS. Furthermore, we wanted to evaluate whether the inflammation-scores added value to well-established prognostic markers as TNM stage and ECOG-PS in lung cancer.

## Methods

### Patients

The cohort has previously been described [20]. In short, in this registry-based cohort study, patients were included if they were diagnosed with lung cancer, registered in The Central Denmark Region and the Danish Lung Cancer Registry between the 1^st^ of January 2009 and 26^th^ of June 2018. The Central Denmark Region has 1,327,410 inhabitants, equivalent to 23% of the Danish population (5,824,857 inhabitants) [21]. In Denmark, all inhabitants are given a unique ten-digit number, the CPR number. The CPR number is used in all public records and allows data to be linked between the various Danish registries and medical records at an individual level. In the Danish Lung Cancer Registry, all primary lung cancer patients have been registered since 2000. The registry has a coverage of more than 90% of all lung cancer patients in Denmark [22]. From the Danish Lung Cancer Registry, the following information was retrieved on each patient at the time of diagnosis: sex, age, ECOG PS, TNM stage, tobacco consumption and histology. Information on tumour histology was confirmed in data retrieved from The national Danish Pathology Data bank [23]. The diagnostic work-up and staging were performed according to the international guideline at the current time. For each patient, data on general inflammation markers were retrieved from the clinical laboratory information system (LABKA) that contain results on all blood samples from hospitalised and outpatients submitted for analyses in the Northern and Central Regions of Denmark [24]. From LABKA, plasma levels of CRP, albumin, haemoglobin, neutrophil count, lymphocyte and monocyte count performed up to 90 days before the lung cancer diagnosis were retrieved. Patients with missing data on one or more of these parameters in the LABKA database were excluded. In the case of more than one measurement, the measurement closest to the diagnosis were extracted. Furthermore, mortality data were retrieved from the Danish Civil Registration System [25].

### Inflammation-scores

The following inflammation-scores were identified, and the following cut points were applied in the study. A high NLR was defined as ≥ 3 [26] and ≥ 4 [27]. The modified Glasgow Prognostic Score (mGPS) were defined as CRP ≤ 8 mg/L and albumin ≥ 35 g/L = score 0; if one of the test results were abnormal = score 1; if both test results were abnormal = score 2 [28]. A high PLR was defined as ≥ 150 [26] and ≥ 200 [29]. The Combined NLR and Glasgow prognostic score (CNG) was defined as score 0 if albumin ≥ 35 g/L, CRP < 8 mg/L and NLR < 2. If one, two or three test results were abnormal, the corresponding score was 1, 2 or 3, respectively [30]. The Aarhus composite biomarker score (ACBS): if albumin ≥ 35 g/L, CRP < 8 mg/L, neutrophil count ≤ 7 × 10^9^ /L, lymphocyte count ≤ 3.5 × 10^9^ /L, and haemoglobin ≥ 7.3(women) / 8.3(men) mmol/L the score 0 was assigned. Conversely, if one, two or three test results were abnormal the corresponding score was 1, 2 or 3, respectively [31]. The HALP score was computed as hemoglobin × albumin × lymphocytes / platelet count. A high HALP was defined as ≥ 26 [32]. A high lymphocyte to monocyte ratio (LMR) was defined as ≥ 2.6 [33] and as high monocyte-to-lymphocyte ratio was defined ≥ 0.367 [34]. The Systemic inflammation index (SII) was calculated by platelet count x neutrophil count / lymphocyte count. A high SII was defined as ≥ 479 [35].

### Ethics

The Danish Patient Safety Authority (no.31–1521-400) and the Danish Data Protection Agency (no. 1–16-02–909-17) have approved the study. According to Danish legislation, registry-based studies do not require approval by the regional committee on health-research ethics. The study was performed in accordance with the Declaration of Helsinki.

### Statistical analysis

Baseline clinicopathological information is presented as numbers and percentages or by the median value with 5% and 95% percentiles. Follow-up time and OS were defined as the time from diagnosis until the death of any cause or last follow-up date (1^st^ of July, 2020), which allowed all patients to be followed for at least two years. Patients still alive on the last day of follow-up were censored. OS was the primary endpoint. Patients with an OS of only one day were excluded. Median OS was estimated by the Kaplan–Meier method and compared by the log-rank test. Crude and adjusted hazard ratios (HR) were calculated by the Cox proportional hazards model. Since only 237 (4%) of the included patients were never-smokers, smoking was not included in the analyses. A directed acyclic graph was analyzed (Supplementary Fig. 1 and supplementary Fig. 2). Confounders were dichotomized except for age, which was analyzed as a continuous variable. A Bonferroni-corrected threshold was applied to account for multiple testing; hence, p-values ≤ 0.003 were considered significant (0.05/17). C-statistics, in terms of Akaike's information criteria (AIC) and Harrell's concordance index (C-index), were calculated to estimate the goodness of fit for the individual inflammation-score. Prognostic models including well-established prognostic markers, TNM stage, age, sex and ECOG PS were compared with models adding the individual inflammation-score. This was done to find the inflammation score with the most accurate prediction of OS. The model with the most precise prediction of OS had the minimum AIC. Only a difference of 2 or more (arbitrary values) was considered an actual difference. For the C-index, values ranged between 0.5 – 1.0, where 1.0 was the perfect fit. Likelihood-ratio tests were used to evaluate whether the added value was significant. The Stata software version 15.1 (Stata Corporation, College Station, Texas, USA) was applied for all statistical analyses. All p-values were two-sided.

## Results

**Fig. 1:**
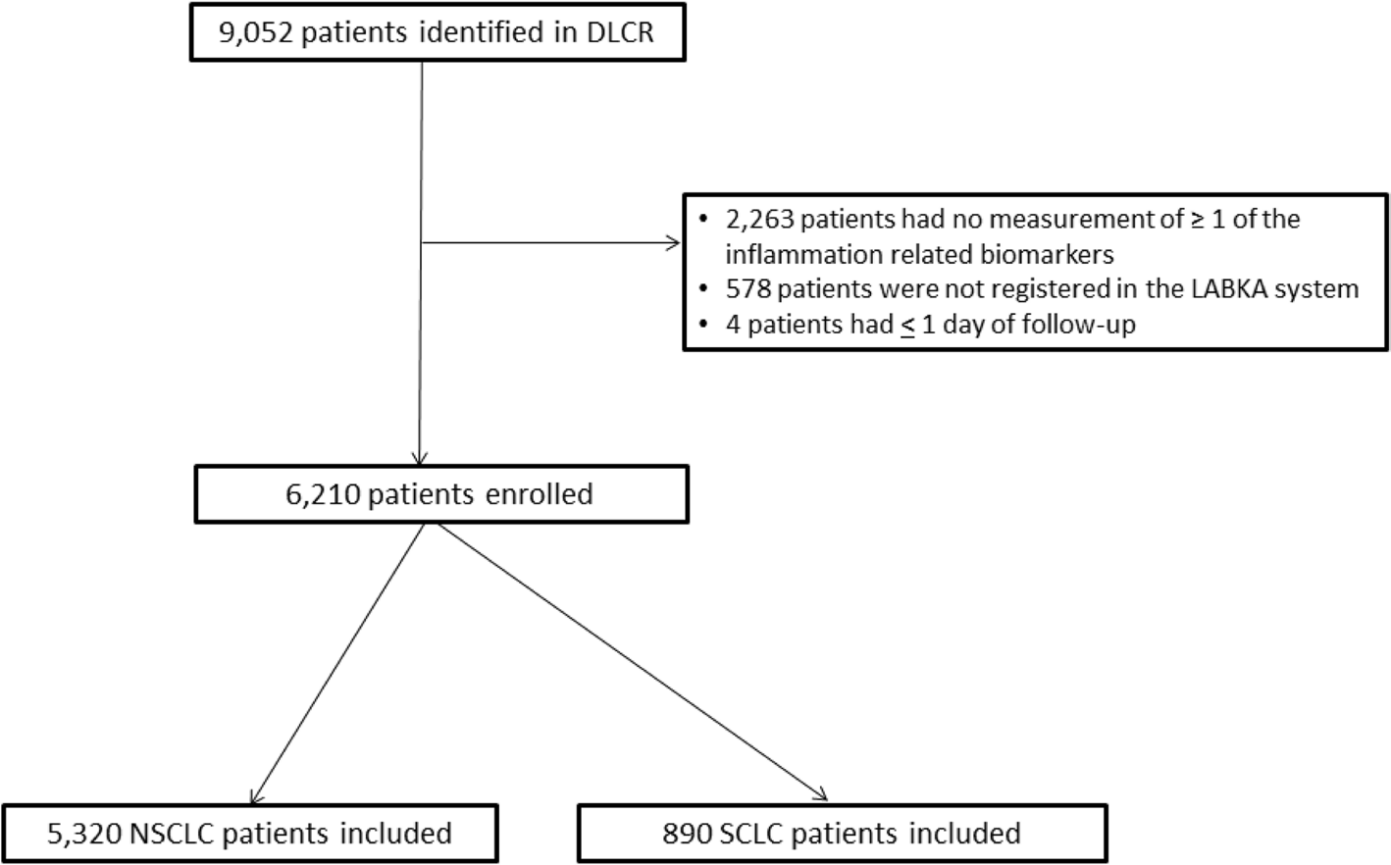
Flow chart of inclusion and exclusion of patients. *DLCR* Danish Lung Cancer Group, *LABKA* clinical laboratory information system, *NSCLC* non-small cell lung cancer, *SCLC* small cell lung cancer, *OS* overall survival

In the Danish Lung Cancer Registry, a total of 9,052 lung cancer patients were identified. Though, 578 of these patients were not registered in the LABKA system and thus excluded. Moreover, four patients with only one day of follow-up and 2,263 patients without one or several of the inflammation-related markers in LABKA were excluded. Hence, 6,210 patients were included in this study (Fig. 1). Patient characteristics for the included and excluded patients are presented in Table 1, demonstrating a similar distribution between the two groups.

**Table 1:** Patient characteristics in all patients included and excluded from the study

	**Excluded from the study** ***N*(%)**	**Included in the study** ***N*(%)**	***P*-value**	**NSCLC** ***N *(%)**	**SCLC** ***N*(%)**
**Total number of patients**	2,263 (31)	6,210 (69)		5,320 (100)	890 (100)
**ECOG PS, N(%)**					
0	784 (35)	1,946 (31)	< 0.0001	1,733 (33)	213 (24)
1	749 (33)	1,894 (31)		1,610 (30)	284 (32)
2	257 (11)	820 (13)		672 (13)	148 (17)
3	297 (13)	854 (14)		704 (13)	150 (17)
NA	176 (8)	696 (11)		601 (11)	95 (10)
** Age, years** median (range 5%—95%)	70 (53 – 85)	70 (52 – 84)	0.06	70 (52 – 84)	69 (52–83)
**Sex**					
Female	1,120 (49)	3,014 (49)	0.44	2,556 (48)	456 (51)
Male	1,143 (51)	3,196 (51)		2,762 (52)	434 (49)
**Stage**					
I	479 (21)	1,188 (19)	< 0.0001	1,142 (21)	46 (5)
II	117 (5)	531 (9)		508 (10)	23 (3)
III	453 (20)	1,167 (19)		943 (18)	224 (25)
IV	1,057 (47)	2,807 (45)		2,291 (43)	518 (58)
NA	107 (5)	517 (8)		436 (8)	79 (9)
**Histology**					
Adenocarcinoma	1,084 (48)	2,769 (45)	0.24	2,769 (52)	
SCC	481 (21)	1,281 (21)		1,281 (24)	
Other	193 (9)	674 (11)		674 (13)	
NSCLC^a^	194 (9)	596 (10)		596 (11)	
SCLC	311 (14)	890 (14)			890 (100)
**Smoking**					
Never Ever NA	114 (5) 1,716 (76) 433 (19)	237 (4) 4,562 (73) 1,411 (23)	< 0.0001	230 (4) 3,899 (73) 1,191 (22)	7 (1) 663 (74) 220 (25)

*P*-value calculated by the Chi-square test or the nonparametric equality of medians tests comparing the included and excluded patients in the study

^a^Not further classified

### Patient characteristics

NSCLC was observed in 5,320 (86%) of all lung cancer patients, and adenocarcinoma was the most frequent subtype in these patients (2,769/5,320) (Table 1). The majority of patients with NSCLC had a good ECOG PS (0–1: 63%), stage IV disease (43%) and a median age of 70 years (5–95% percentiles: 52–84). At the time of follow-up, 4,259 of the 5,320 (92%) patients had died. The median follow-up was 0.82 years (5–95% percentiles: 0.3–7.3 years).

Similar observations were made in the 890 patients with SCLC included in the study. Here, the majority of patients had a good ECOG PS (0–1:56%), stage IV disease (58%) and a median age of 69 years (5–95% percentiles: 52–83 years) (Table 1). At the time of follow-up, 831 of the 890 (93%) had died. The median follow-up was 0.62 years (5–95% percentiles:0.02–5.5 years).

### Inflammation-scores and survival

The distribution of general inflammation biomarkers and their crude association with OS in NSCLC and SCLC patients is presented in Supplementary table 1.

For all the composed inflammation-scores, a higher score was associated with an inferior OS in patients with NSCLC, except for LMR, where a lower score was associated with inferior OS, and for HALP where a correlation to OS could not be detected (Table 2). When adjusting for confounders, all inflammation-scores except the HALP score remained significant as prognostic biomarkers of OS.

**Table 2:** Inflammation-scores association with overall survival in NSCLC

	**Cut points**	***N*(%)**	**Univariate HR (95%CI)**	***P*-value**	**Adjusted HR (95%CI)**	***P*-value**
NLR	< 3	1,658 (31)	1.00		1.00	
	> 3	3,662 (69)	2.17 (2.02 – 2.32)	< 0.0001	1.52 (1.40 – 1.64)	< 0.0001
	< 4	2,534 (48)	1.00		1.00	
	> 4	2,786 (52)	2.21 (2.07 – 2.35)	< 0.0001	1.58 (1.47 – 1.69)	< 0.0001
mGPS	0	1,498 (30)	1.00		1.00	
	1	1,883 (36)	2.04 (1.88 – 2.21)	< 0.0001	1.43 (1.30 – 1.57)	< 0.0001
	2	1,939 (35)	2.95 (2.72 – 3.20)	< 0.0001	1.70 (1.55 – 1.86)	< 0.0001
PLR	< 150	1,571 (30)	1.00		1.00	
	> 150	3,749 (70)	1.61 (1.50 – 1.73)	< 0.0001	1.25 (1.15 – 1.34)	< 0.0001
	< 200	2,645 (50)	1.00		1.00	
	> 200	2,675 (50)	1.79 (1.68 – 1.90)	< 0.0001	1.32 (1.23 – 1.42)	< 0.0001
CNG	0	329 (5)	1.00		1.00	
	1	1,367 (27)	1.46 (1.24 – 1.72)	< 0.0001	1.25 (1.05 – 1.49)	0.012
	2	1,809 (34)	2.86 (2.44 – 3.35)	< 0.0001	1.74 (1.47 – 2.06)	< 0.0001
	3	1,815 (34)	4.18 (3.57 – 4.89)	< 0.0001	2.10 (1.77 – 2.49)	< 0.0001
ACBS	0	666 (13)	1.00		1.00	
	1	985 (19)	1.58 (1.39 – 1.80)	< 0.0001	1.42 (1.24 – 1.64)	< 0.0001
	2	1,067 (20)	2.30 (2.03 – 2.61)	< 0.0001	1.60 (1.40 – 1.83)	< 0.0001
	3	2,602 (49)	3.97 (3.54 – 4.44)	< 0.0001	2.24 (1.97 – 2.54)	< 0.0001
HALP	< 26	5,209 (98)	1.00		1.00	
	> 26	111 (2)	0.95 (0.78 – 1.17)	0.639	1.28 (1.03 – 1.61)	0.029
LMR	< 2.6	3,345 (63)	1.00		1.00	
	> 2.6	1,975 (37)	0.57 (0.53 – 0.61)	< 0.0001	0.78 (0.72 – 0.84)	< 0.0001
MLR	< 0.367	1,811 (34)	1.00		1.00	
	> 0.367	3,509 (64)	1.75 (1.63 – 1.87)	< 0.0001	1.31 (1.21 – 1.41)	< 0.0001
SII	< 479	551 (10)	1.00		1.00	
	> 479	4,769 (90)	1.79 (1.61 – 2.00)	< 0.0001	1.34 (1.19 – 1.51)	< 0.0001

*ACBS* Aarhus composite biomarker score, *CI* confidence interval, *CNG* The Combined *NLR* and Glasgow prognostic score, *HALP* haemoglobin × albumin × lymphocytes/platelet count, *HR* hazard ratio, *LMR* lymphocyte to monocyte ratio, *mGPS* modified Glasgow prognostic score, *MLR* Monocyte to lymphocyte ratio, *NLR* Neutrophil to lymphocyte ratio, *NSCLC* Non-small cell lung cancer, *PLR* thrombocyte to lymphocyte ratio *SII* The systemic inflammation index

*P*-value calculated by the Log likelihood test

In SCLC, seven of the nine evaluated scores were associated with an inferior OS: NLR, mGPS, PLR, CNG, ACBS, LMR and MLR (Table 3). After adjusting for confounders, they all remained significant as prognostic biomarkers of OS (Table 3).

**Table 3:** Inflammation-scores association with overall survival in SCLC

	**Cut points**	***N*(%)**	**Univariate HR (95%CI)**	***P*-value**	**Adjusted HR (95%CI)**	***P*-value**
NLR	< 3	286 (32)	1.00		1.00	
	> 3	604 (68)	1.64 (1.41 – 1.90)	< 0.0001	1.35 (1.15 – 1.60)	< 0.0001
	< 4	417 (47)	1.00		1.00	
	> 4	473 (53)	1.78 (1.55 – 2.06)	< 0.0001	1.52 (1.30 – 1.77)	< 0.0001
mGPS	0	255 (29)	1.00		1.00	
	1	365 (41)	1.36 (1.15 – 1.62)	< 0.0001	1.26 (1.04 – 1.52)	0.017
	2	270 (30)	2.13 (1.77 – 2.55)	< 0.0001	1.67 (1.35 – 2.05)	< 0.0001
PLR	< 150	281 (32)	1.00		1.00	
	> 150	609 (68)	1.10 (0.95 – 1.28)	0.211	1.08 (0.92 – 1.27)	0.360
	< 200	457 (51)	1.00		1.00	
	> 200	433 (49)	1.22 (1.06 – 1.40)	0.005	1.19 (1.03 – 1.39)	0.021
CNG	0	45 (5)	1.00		1.00	
	1	262 (29)	1.90 (1.32 – 2.74)	0.001	1.65 (1.10 – 2.49)	0.018
	2	328 (37)	2.23 (1.56 – 3.20)	< 0.0001	1.88 (1.25 – 2.82)	0.003
	3	255 (29)	3.40 (2.36 – 4.91)	< 0.0001	2.55 (1.68 – 3.87)	< 0.0001
ACBS	0	92 (10)	1.00		1.00	
	1	174 (20)	1.50 (1.14 – 1.98)	0.004	1.61 (1.19 – 2.19)	0.002
	2	218 (24)	1.70 (1.30 – 2.22)	< 0.0001	1.45 (1.08 – 1.95)	0.014
	3	406 (46)	2.41 (1.88 – 3.09)	< 0.0001	1.98 (1.50 – 2.62)	< 0.0001
HALP	< 26	864 (97)	1.00		1.00	
	> 26	26 (3)	1.07 (0.73 – 1.59)	0.724	1.00 (0.65 – 1.54)	0.983
LMR	< 2.6	508 (57)	1.00		1.00	
	> 2.6	382 (43)	0.74 (0.64 – 0.85)	< 0.0001	0.80 (0.69 – 0.93)	0.005
MLR	< 0.367	354 (40)	1.00		1.00	
	> 0.367	536 (60)	1.28 (1.11 – 1.47)	0.001	1.23 (1.05 – 1.43)	0.010
SII	< 479	107 (12)	1.00		1.00	
	> 479	783 (88)	1.10 (0.88 – 1.36)	0.405	1.13 (0.89 – 1.44)	0.326

*ACBS* Aarhus composite biomarker score, *CI* confidence interval, *CNG* The Combined *NLR* and Glasgow prognostic score, *HALP* haemoglobin × albumin × lymphocytes/platelet count, *HR* hazard ratio, *LMR* lymphocyte to monocyte ratio, *mGPS* modified Glasgow prognostic score, *MLR* Monocyte to lymphocyte ratio, *NLR* Neutrophil to lymphocyte ratio, *PLR* thrombocyte to lymphocyte ratio, *SCLC *Small cell lung cancer, *SII* The systemic inflammation index

*P*-value calculated by the Log likelihood test

### Comparisons of inflammation-scores

In NSCLC patients, the ACBS and NLR were the inflammation-scores with the best prognostication of OS when evaluated individually (Table 4). Furthermore, all models, including an inflammation-score, except for the model including HALP, improved the AIC and C-index compared to the model only including the established prognostic markers (Table 4). Moreover, the ACBS and NLR remained the inflammation-scores with the best prognostication of OS compared to the other inflammation-scores. Both scores significantly improved the model fit (*p* < 0.0001), illustrating that the scores significantly added value to the well-established prognostic markers alone.

**Table 4:** Predictive Accuracies of the Prognostic Models

	**NSCLC**		**SCLC**	
**Model**	**AIC**	**C-index**	**AIC**	**C-index**
NLR^a^	65,397	0.594	9,622	0.559
NLR^b^	65,286	0.612	9,600	0.575
mGPS	65,175	0.635	9,602	0.588
PLR^c^	65,722	0.556	9,664	0.506
PLR^d^	65,572	0.579	9,657	0.522
CNG	65,055	0.646	**9,599**	0.589
ACBS	**64,952**	**0.651**	9,604	**0.594**
HALP	65,917	0.502	9,665	0.501
LMR	65,529	0.576	9,647	0.547
MLR	65,630	0.574	9,653	0.543
SII	65,786	0.533	9,665	0.504
Stage + age + sex + PS + smoking	52,232	0.769	7,743	0.730
NLR^a^ + stage + age + sex + PS + smoking	52,118	0.775	7,732	0.731
NLR^b^ + stage + age + sex + PS + smoking	**52,065**	**0.777**	**7,717**	**0.736**
mGPS + stage + age + sex + PS + smoking	52,100	0.776	**7,718**	**0.737**
PLR^c^ + stage + age + sex + PS + smoking	52,201	0.770	7,744	0.730
PLR^d^ + stage + age + sex + PS + smoking	52,169	0.771	7,739	0.731
CNG + stage + age + sex + PS + smoking	52,075	0.778	**7,717**	**0.737**
ACBS + stage + age + sex + PS + smoking	**52,019**	**0.779**	7,720	0.737
HALP + stage + age + sex + PS + smoking	52,230	0.769	7,745	0.730
LMR + stage + age + sex + PS + smoking	52,105	0.771	7,737	0.732
MLR + stage + age + sex + PS + smoking	52,182	0.771	7,738	0.732
SII + stage + age + sex + PS + smoking	52,210	0.770	7,744	0.730

*ACBS* Aarhus composite biomarker score, *AIC* Akaikes information criterion, *CNG* The Combined NLR and Glasgow prognostic score, *HALP* haemoglobin × albumin × lymphocytes/platelet count, *LMR* lymphocyte to monocyte ratio, *mGPS* modified Glasgow prognostic score, *MLR* Monocyte to lymphocyte ratio, *NLR* Neutrophil to lymphocyte ratio, *NSCLC* non-small cell lung cancer, *PLR* thrombocyte to lymphocyte ratio, *SCLC* Small cell lung cancer, *SII* The systemic inflammation index^a^ cut point ≥ 3; ^b^ cut point ≥ 4; ^c^ cut point ≥ 150; ^d^ cut point ≥ 150 AIC estimates the quality of each model relative to the other models. The values are arbitrary. The model with the minimum, *AIC* is the model with the optimal fit of data. ** c-index: Harrell's concordance index. C-index gives a measure of goodness of fit for the model. Values range between 0.5 – 1.0; 1.0 is the perfect fit

In SCLC patients, the CNG was the individual inflammation-score with the optimal model fit based on the AIC, whereas the ACBS had the optimal model fit based on the C-index (Table 4). When evaluating the models, three inflammation-scores, NLR, mGPS and CNG, had superior prognostication of OS compared to the other inflammation-scores based on the AIC and C-index. All three scores significantly improved the model fit (*p* < 0.0001) and added value to the well-established prognostic markers alone.

## Discussion

In this registry-based study of 6,210 lung cancer patients, we evaluated nine inflammation-scores assessed previously in lung cancer. In 5,320 patients with NSCLC, we found that all the assessed inflammation-scores except HALP could predict OS. In contrast, only NLR, mGPS, CNG, ACBS and LMR could significantly predict OS in the 890 patients with SCLC. Furthermore, we made a direct comparison of the inflammation-scores and found that the ACBS and NLR were the most optimal scores to predict an inferior OS in patients with NSCLC while three scores, NLR, mGPS and CNG, were of equal superiority in patients with SCLC.

The nine evaluated inflammation-scores have all previously shown potentials as prognostic biomarkers of OS in lung cancer patients [26–29, 32, 34–38]. NLR is the most widely studied score and has in meta-analyses demonstrated to hold prognostic value in NSCLC [39–41] as well as in SCLC [17, 19]. On the other hand, the ACBS has only once been studied in patients with lung cancer as we previously have evaluated the score in 267 patients with NSCLC. Here, the score was found prognostic of OS and to hold higher prognostic value than the NLR score [38].

For prognostic markers to be established in the everyday clinic, they have to be accessible, reproducible, and most importantly, aid the clinician counselling the patient. Therefore, inflammation-scores based on general inflammation markers have drawn attention since they are readily available. The reproducibility of these routine blood tests, in general, is very high. However, whether these scores aid the treating clinician has not yet been systematically evaluated in a large patient cohort. By employing c-statistics in terms of Harrel's C-index and AIC, we demonstrated the inflammation-scores add value in the prognostication of OS. Thus, our findings indicate that inflammation-scores could be useful markers when applied with established prognostic markers to define a subset of lung cancer patients with inferior prognosis requiring a more intensive treatment. Furthermore, based on c-statistics, we identified ACBS and NLR as the superior inflammation-scores in NSCLC patients and NLR, mGPS and CNG as superior scores in SCLC patients. However, in SCLC patients, the ACBS had an equivalent high C-index indicating that the ACBS could be non-inferior to the NLR, mGPS and CNG. Harrel's C-index and AIC are both measures of model fit for logistic regression but measuring slightly different aspects of the models [42, 43]. In time-to-event statistics, Harrel's C-index is based on the area under the curve calculations at multiple specific time points risking different conclusions at different time points. In contrast, the AIC is based on the information-theoretic framework instead of a null hypothesis testing [44]. Therefore, when non-consistent measures of AIC and C-index were observed, the AIC overruled.

The biological mechanisms underlying the prognostic value of inflammation-scores have not yet been revealed. The pro-tumorigenic function of interleukin-1 is widely accepted, just as it is established that the pro-inflammatory cytokine interleukin-1β plays an imperative role in sustaining the tumour microenvironment leading to tumour genesis and progression [45]. In clinical studies, interleukin-1β has been linked to cancer frequency, and mortality since inhibition of the cytokine led to decreased lung cancer incidence and mortality in atherosclerotic patients [46]. Additionally, in rheumatic patients, high levels of interleukin-1β were associated with anaemia, neutrophilia, lymphopenia, low albumin levels, and increased CRP [47], which are elements partly used in several of the inflammation-scores. Hence, it is likely that the inflammation-scores are reflections of interleukin-1β. Thus, the inflammation-score with the most optimal reflection of interleukin-1β would, in theory, be the best prediction of survival. The ACBS encompasses both neutrophilia and lymphopenia, along with anaemia and CRP, and would thus be expected as the best reflection of the IL-1β. In line with this, the ACBS was the best performing inflammation-scores in NSCLC patients in this study. Nevertheless, in patients with SCLC, we found that the value of NLR, mGPS and CNG was superior to the ACBS, even though they only reflect two or three parameters influenced by interleukin-1β.

This study's main advantage is the magnitude of the dataset, which, to our knowledge, makes it the most extensive cohort study of inflammation-scores in lung cancer patients. When comparing several prognostic markers using C-statistics, a large cohort like ours is needed to assure the statistical power [48]. Furthermore, we retrieved information from the Danish public healthcare system and the Danish Lung Cancer Register, which has very high completeness [22], allowing population-based studies of all lung cancer patients independently of economic or social differences. Owed to the Danish administrative system, we included all lung cancer patients from a well-defined geographical area, reducing the risk of selection bias to a minimum. However, despite the study's largeness and the completeness of data, there are some limitations to consider. We did not have data on comorbidity, which could affect the inflammation-scores. However, as the association between comorbidity and increased risk of death in lung cancer patients still is debated [49] and no data exist on whether the index is associated with any of the evaluated inflammation scores, we believe that the lack of comorbidity data is a limitation that can be accepted in this study. Although the Danish registers have high completeness, we observed a high number of missing data in our dataset as we only included patients where all the inflammation-scores could be evaluated. Consequently, we had to exclude 2263 patients with one or more of the inflammation-related markers missing. As this is a retrospective study of real-time data, variations in the number of available blood samples from each patient were expected. However, by excluding a large number of patients, an unintended bias may have emerged. Yet, we performed a comparison of clinical parameters between the in- and excluded patients in the study and did not find indications of a bias. Moreover, we did not have information on the mutational status of any molecular targets, for example, epidermal growth factor receptor, and, therefore, we cannot rule out that a potential bias could be present. Lastly, the lack of data on treatment in this study could be a limitation as targeted therapies and immune therapies have changed practice during the inclusion period. This is known to have improved OS over the last decades, however, this have not been included in this study and may give biased results. However, we have performed timesplit analyses and observed that the ranking of the inflammation-scores remain unaffected.

In conclusion, nine inflammation-scores were evaluated in patients with NSCLC and eight of the evaluated scores were prognostic markers of OS. Similarly, seven of the nine evaluated scores were prognostic of OS in patients with SCLC. Furthermore, all inflammation-scores improved the prognostic value of well-established prognostic markers. Still, the ACBS and NLR were the superior inflammation-scores in NSCLC, whereas the NLR, mGPS and CNG were superior in SCLC. These results indicate that inflammation-scores could add value to the general prognostication of lung cancer patients in the future.

## Supplementary Information

**Additional file 1**.

**Additional file 2**.  

**Additional file 3**. **Table 1** The individual inflammation markers association with overall survival  

## Data Availability

The data that support the findings of this study are available from Danish Lung Cancer Registry, The national Danish Pathology Data bank and the clinical laboratory information system, administered by The Central Denmark Region, but restrictions apply to the availability of these data, which were used under license for the current study, and so are not publicly available. Data are however available from the authors upon reasonable request and with permission of The Central Denmark Region. Ethics approval and consent to participate: The Danish Patient Safety Authority (no.31–1521-400) and the Danish Data Protection Agency (no. 1–16-02–909-17) have approved the study. According to Danish legislation, registry-based studies do not require approval by the regional committee on health-research ethics. The study was performed in accordance with the Declaration of Helsinki.

## Abbreviations

ECOG PS: Eastern cooperative oncology group performance status
N: Number
NA: Non-available
NSCLC: Non-small cell lung cancer
SCLC: Small cell lung cancer
SCC: Squamous-cell carcinoma
