# Supplementary figures and images for: Inflammation-scores as prognostic markers of overall survival in lung cancer: a register-based study of 6,210 Danish lung cancer patients
Source: BMC Cancer. 2022 Jan 14;22:63. doi: 10.1186/s12885-021-09108-5 (PMC8759208; doi:10.1186/s12885-021-09108-5)

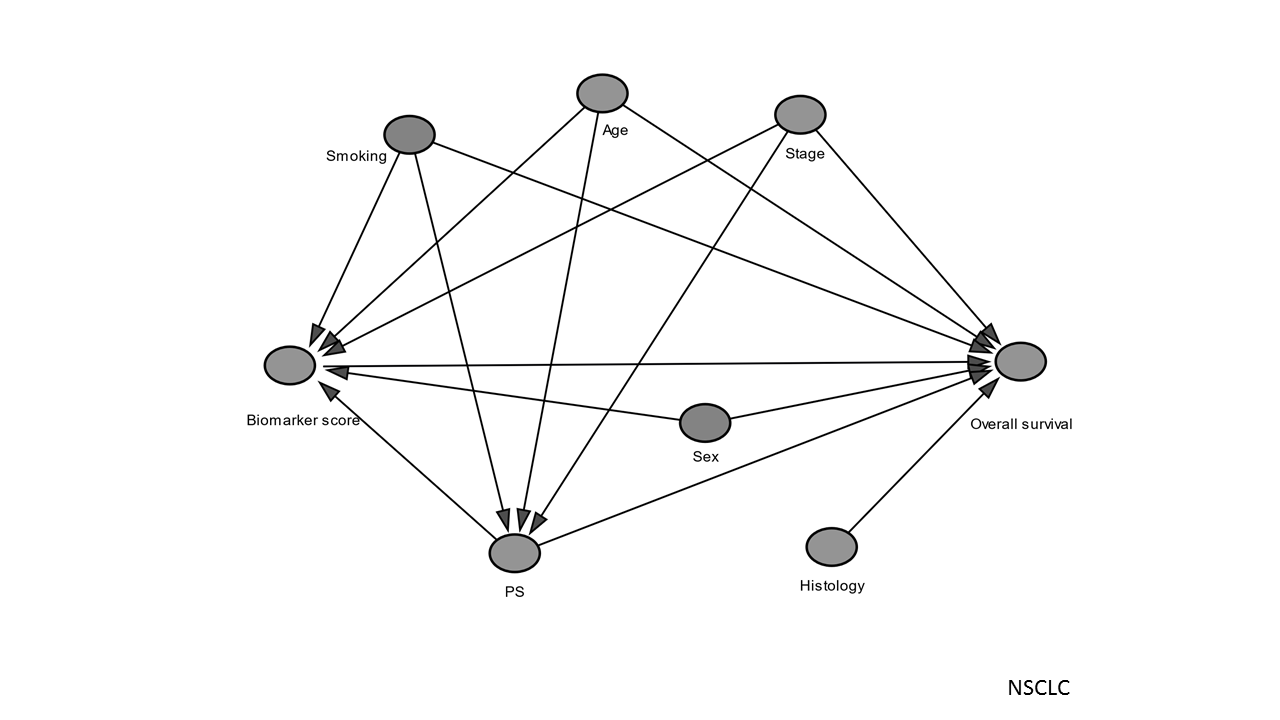

Supplement: Supplementary file 1 — Additional file 1. [file 12885_2021_9108_MOESM1_ESM.tif]

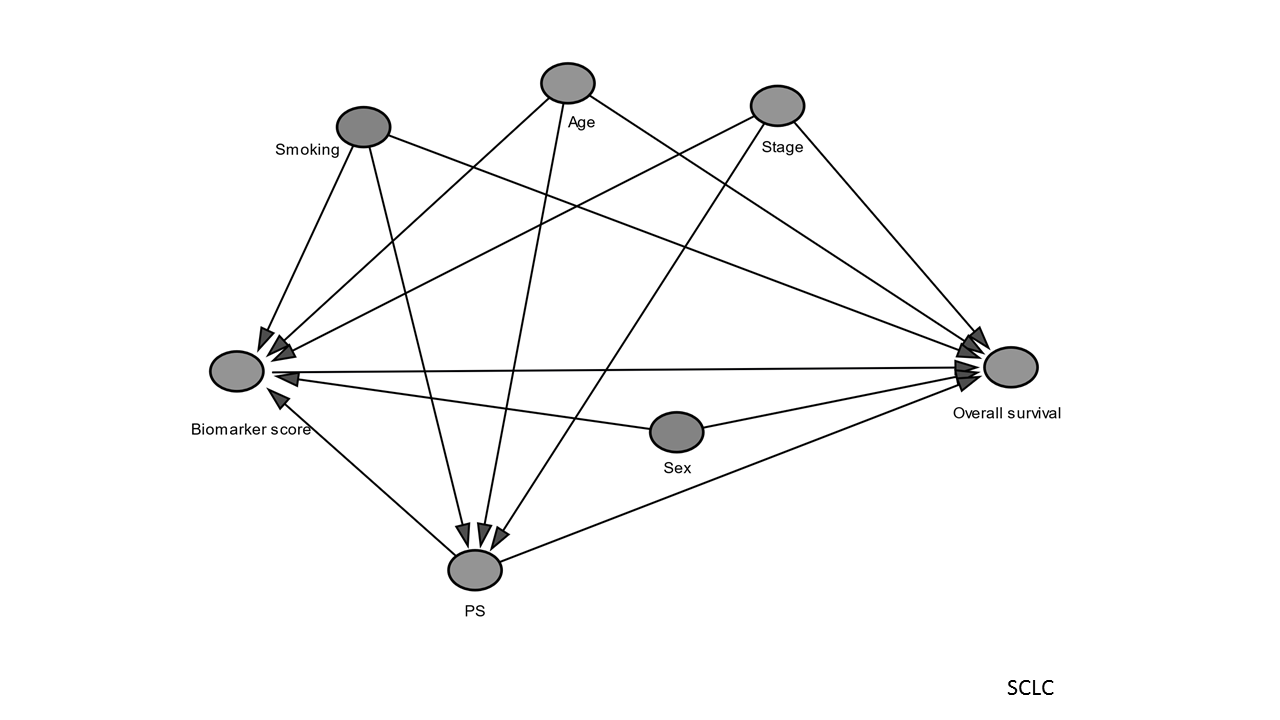

Supplement: Supplementary file 2 — Additional file 2. [file 12885_2021_9108_MOESM2_ESM.tif]
