# Supplementary material for: Inflammation-scores as prognostic markers of overall survival in lung cancer: a register-based study of 6,210 Danish lung cancer patients
Source: BMC Cancer. 2022 Jan 14;22:63. doi: 10.1186/s12885-021-09108-5 (PMC8759208; doi:10.1186/s12885-021-09108-5)
Supplement: Supplementary file 3 — Additional file 3. Table 1 The individual inflammation markers association with overall survival [file 12885_2021_9108_MOESM3_ESM.docx]

Supplementary table 1. The individual inflammation markers association with overall survival

|  |  | **NSCLC** | | | **SCLC** | | |
| --- | --- | --- | --- | --- | --- | --- | --- |
| **Biomarker** | **Cut point** | **N (%)** | **Univariate**  **HR (95% CI)** | **p-value** | **N (%)** | **Univariate**  **HR (95%CI)** | **p-value** |
| Albumin | ≥ 34 g/L | 3,299 (62) |  |  | 593 (67) |  |  |
|  | < 34 g/L | 2,021 (38) | 1.92 (1.81 – 2.04) | <0.0001 | 297 (33) | 1.76 (1.52 – 2.04) | <0.0001 |
| Hgb | ≥ 7.3^w^ / 8.3^m^ mmol/L | 3,247 (61) |  |  | 597 (67) |  |  |
|  | < 7.3^w^ / 8.3^m^ mmol/L | 2,073 (39) | 1.60 (1.50 – 1.69) | <0.0001 | 293 (33) | 1.20 (1.04 – 1.40) | 0.013 |
| CRP | ≤ 10 mg/L | 1,877 (35) |  |  | 340 (38) |  |  |
|  | > 10 mg/L | 3,443 (65) | 2.23 (2.09 – 2.39) | <0.0001 | 550 (62) | 1.44 (1.25 – 1.66) | <0.0001 |
| Neutrophil count | ≤ 7.0 × 10^9^ /L | 2,949 (55) |  |  | 489 (55) |  |  |
|  | >7.0 × 10^9^ /L | 2,371 (45) | 1.97 (1.86 – 2.10) | <0.0001 | 401 (45) | 1.55 (1.35 – 1.78) | <0.0001 |
| Lymphocyte count | ≤3.5 × 10^9^ /L | 5,149 (97) |  |  | 862 (97) |  |  |
|  | >3.5 × 10^9^ /L | 171 (3) | 0.65 (0.54 – 0.78) | <0.0001 | 28 (3) | 0.89 (0.60 – 1.32) | 0.560 |
| Monocyte count | ≤0.7 × 10^9^ /L | 2,531 (48) |  |  | 478 (54) |  |  |
|  | >0.7 × 10^9^ /L | 2,789 (52) | 1.23 (1.16 – 1.31) | <0.0001 | 412 (46) | 1.07 (0.93 – 1.22) | 0.369 |
| Platelet count | ≤ 400^w^ / 350^m^ × 10^9^ /L | 3,802 (71) |  |  | 670 (75) |  |  |
|  | >400^w^ / 350^m^ × 10^9^ /L | 1,518 (29) | 1.38 (1.30 – 1.48) | <0.0001 | 220 (25) | 1.08 (0.92 – 1.26) | 0.890 |

CI: confidence interval; CRP: c-reactive protein; Hgb: haemoglobin; HR: hazard ratio; NSCLC: Non-small cell lung cancer; SCLC: Small cell lung cancer

^w^women; ^m^men;

P-value calculated by the Log likelihood test.
